# Editorial

**DOI:** 10.3164/jcbn.67-2_Editorial

**Published:** 2020-08-06

**Authors:** Yuji Naito

**Affiliations:** 1 Executive Editor of JCBN; 2 Kyoto Prefectural University of Medicine

Hope you and your family are safe and fighting against #COVID-19. By the end of June, the corona virus SARS-CoV-2 had infected more than 11 million people and killed more than 500,000 people in more than 150 countries. Fortunately, Japan was able to contain the first attack of COVID-19, but in July, the second infection trend has begun. The battle against SARS-CoV-2 is expected to be long-term, and the accumulation of scientific knowledge is extremely important for solving many difficult problems. This month’s issue of JCBN published four papers on COVID-19.

Wei *et al.*^(1)^ evaluated the nutritional status in 348 severe patients with COVID-19 in China, and demonstrated that the CONUT score independently predicts the prognosis of COVID-19 patients, which can help physicians to clarify patients with poor prognosis. The CONUT score was calculated from lymphocyte counts, total cholesterol and serum albumin levels (Table 1). Their data indicate that malnutrition is significantly associated with poor outcome of COVID-19, while the prognosis of patients with normal nutrition status is relatively favorable.

Lin *et al.*^(2)^ identified the most useful prognostic factors to predict critical illness incidence from severe COVID-19 patients who were treated at the intensive care unit in China. They found that hypoproteinemia was an independent risk factor associated with deterioration of severe patients, the cutoff of 29.6 g/L.

Mori *et al.*^(3)^ proposed the statements regarding high-resolution manometry (HRM), a useful clinical test for gastrointestinal motility, in consideration of the current situation of COVID-19, especially focusing on the safety of medical staff and subjects. Similar policies should be followed for HRM that is considered to have the same risk of infection as endoscopy.

Finally, we demonstrated that the number of deaths from COVID-19 infections increased in clear proportion to the number of infected patients, and there is no significant difference in the mortality rate among countries.^(4) ^We suggested that the reason for the low number of deaths from COVID-19 in Japan is probably due to the low infection rate per population. In addition, we showed that there was a strong positive correlation between the frequency of selective IgA deficiency and the COVID-19 infection rate per population.

JCBN will continue to widely accept papers on COVID-19 and make efforts to provide information promptly. I look forward to enjoying many papers from everyone.

## Figures and Tables

**Table 1:** The evaluation of malnutrition by the CONUT score^(1)^

	Range and Score			
Albumin (g/dl)	≥3.50	3.00–3.49	2.50–2.99	<2.50
Score	0	2	4	6
Total cholesterol (mg/dl)	≥180	140–179	100–139	<100
Score	0	1	2	3
Lymphocyte counts (/mm^3^)	≥1,600	1,200–1,599	800–1,199	<800
Score	0	1	2	3

The CONUT score was calculated from serum albumin levels, total cholesterol and lymphocyte counts.
